# COVID-19 predictability in the United States using Google Trends time series

**DOI:** 10.1038/s41598-020-77275-9

**Published:** 2020-11-26

**Authors:** Amaryllis Mavragani, Konstantinos Gkillas

**Affiliations:** 1 Department of Computing Science and Mathematics, Faculty of Natural Sciences, University of Stirling, Stirling, FK9 4LA Scotland, UK; 2 Department of Management Science and Technology, University of Patras, Patras, Greece

**Keywords:** Infectious diseases, Public health, Epidemiology, Bioinformatics, Statistical methods

## Abstract

During the unprecedented situation that all countries around the globe are facing due to the Coronavirus disease 2019 (COVID-19) pandemic, which has also had severe socioeconomic consequences, it is imperative to explore novel approaches to monitoring and forecasting regional outbreaks as they happen or even before they do so. To that end, in this paper, the role of Google query data in the predictability of COVID-19 in the United States at both national and state level is presented. As a preliminary investigation, Pearson and Kendall rank correlations are examined to explore the relationship between Google Trends data and COVID-19 data on cases and deaths. Next, a COVID-19 predictability analysis is performed, with the employed model being a quantile regression that is bias corrected via bootstrap simulation, i.e., a robust regression analysis that is the appropriate statistical approach to taking against the presence of outliers in the sample while also mitigating small sample estimation bias. The results indicate that there are statistically significant correlations between Google Trends and COVID-19 data, while the estimated models exhibit strong COVID-19 predictability. In line with previous work that has suggested that online real-time data are valuable in the monitoring and forecasting of epidemics and outbreaks, it is evident that such infodemiology approaches can assist public health policy makers in addressing the most crucial issues: flattening the curve, allocating health resources, and increasing the effectiveness and preparedness of their respective health care systems.

## Introduction

In December 2019, a novel coronavirus of unknown source was identified in a cluster of patients in the city of Wuhan, Hubei, China^1^. The outbreak first came to international attention after the World Health Organization (WHO) reports said that there was a cluster of pneumonia cases on Twitter on January 4th^2^, followed by the release of an official report on January 5th^3^. China reported its first COVID-19-related death on January 11th, while on January 13th, the first case outside China was identified^4^. On January 14th, the World Health Organization (WHO) tweeted that Chinese preliminary investigations reported that no human-to-human transmission had been identified^5^. However, the virus quickly spread to other Chinese regions and neighboring countries, while Wuhan, identified as the epicenter of the outbreak, was cut off by authorities on January 23rd, 2020^6^. On January 30th, the WHO declared the epidemic to be a public health emergency^1^, and the disease caused by the virus received its official name, that is, COVID-19, on February 11th^7^.

**Figure 1:**
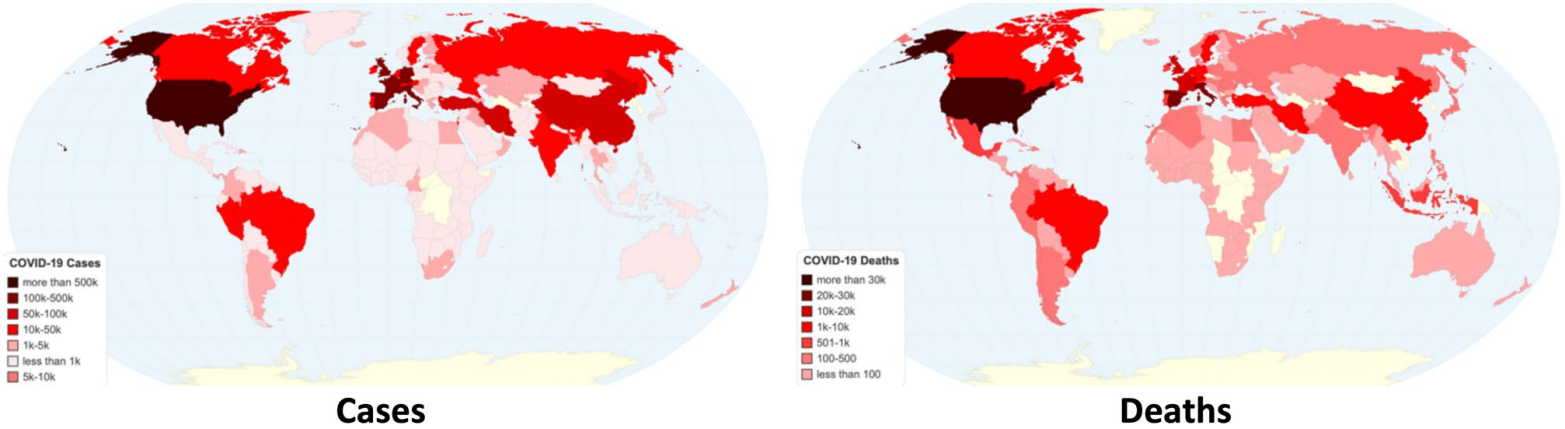
Geographical distribution of worldwide COVID-19 cases and deaths as of April 18th (Chartsbin^43^).

The first serious COVID-19 outbreak in Europe was identified in northern Italy during February, with the country recording its first death on February 21st^8^. The novel coronavirus was transmitted to all parts of Europe within the next few weeks, and as a result, the WHO declared COVID-19 to be a pandemic on March 11th, 2020. As of 16:48 GMT on April 18th, 2020^9^, there were 2,287,369 confirmed cases worldwide, with 157,468 confirmed deaths and 585,838 recovered patients. The most affected countries with more than 100 k cases (in absolute numbers, not divided by population) were the US, with 715,105 confirmed cases and 37,889 deaths; Spain, with 191,726 confirmed cases and 20,043 deaths; Italy, with 175,925 confirmed cases and 23,227 deaths; France, with 147,969 confirmed cases and 18,681 deaths; Germany, with 142,614 confirmed cases and 4405 deaths; and the UK, with 114,217 confirmed cases and 15,464 deaths. The worldwide geographical distribution of COVID-19 cases and deaths by country is depicted in Fig. 1.

**Figure 2:**
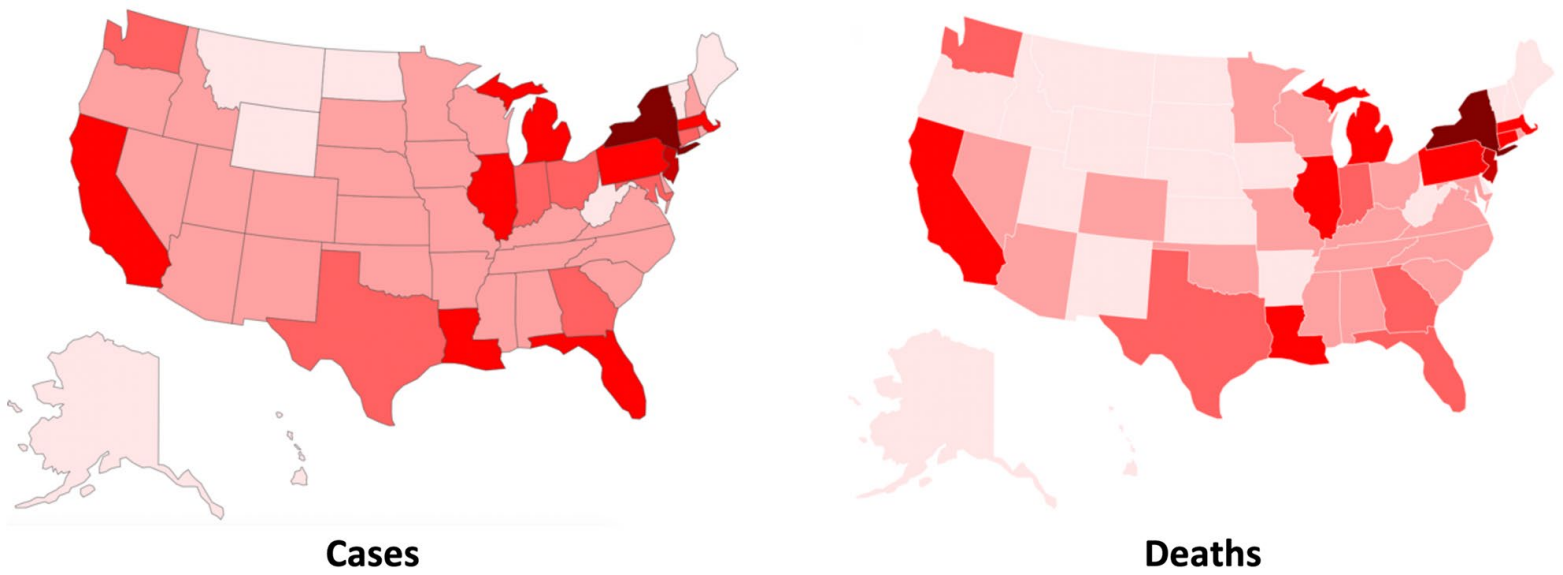
Geographical distribution of COVID-19 cases and deaths in the US as of April 18th (Pixelmap^42^).

As shown, Europe has been severely affected by COVID-19. However, the spread of the disease now indicates that the center of the epidemic has moved to the US, with the state of New York counting more than 240 k cases and 17 k deaths. Figure 2 shows the distribution of COVID-19 cases and deaths in the United States by state as of April 18th, 2020^10^.

To find new methods and approaches for disease surveillance, it is crucial to take advantage of real-time internet data. Infodemiology, i.e., information epidemiology, is a concept that was introduced by Gunther Eysenbach^11,12^. In the field of infodemiology, internet sources and data are employed to inform public health and policy^13,14^. These approaches have been suggested to be valuable for the monitoring and forecasting of outbreaks and epidemics^15^, such as Ebola^16^, Zika^17^, MERS^18^, influenza^19^, and measles^20,21^.

During the COVID-19 pandemic, several research studies using web-based data have been published. Google Trends, the most popular infodemiology source along with Twitter, has been widely used in health and medicine for the analysis and forecasting of diseases and epidemics^22^. As of April 20, 2020, seven (7) papers on the topic of monitoring, tracking, and forecasting COVID-19 using Google Trends data had already appeared online in PubMed (advanced search: covid AND google trends)^23^ for several regions: Taiwan^24^, China^25,26^, Europe^27,28^, the US^28,29^, and Iran ^28,30^. Note that for Twitter publications related to the COVID-19 pandemic, eight papers (8) published from March 13, 2020 to April 20, 2020^31–38^ are available online (PubMed advanced search: covid AND twitter^23^). Table 1 systematically reports these COVID-19 Google Trends studies, in order of the reported publication date.

**Table 1:** Systematic reporting of publications on COVID-19 using Google Trends as of April 20th, 2020.

Authors	Date	Region	Objective	Publisher	Journal
Husnayain et al.^24^	March 12	Taiwan	Analyzing COVID-19 related searches	Elsevier	International Journal of Infectious Diseases
Li et al.^25^	March 25	China	Correlating Internet searches with COVID-19 cases	Eurosurveillance	Eurosurveillance
Mavragani^27^	April 2	Europe	Correlating Google Trends data with COVID-19 cases and deaths	JMIR	JMIR Public Health and Surveillance
Hong et al.^29^	April 7	USA	Relationship between telehealth searches and COVID-19	JMIR	JMIR Public Health and Surveillance
Walker et al.^28^	April 11	USA, Iran, Europe	Exploring of the online activity related to loss of smell	Wiley	International Forum of Allergy and Rhinology
Ayyoubzadeh et al.^30^	April 14	Iran	Prediction of COVID-19 cases	JMIR	JMIR Public Health and Surveillance
Effenberger et al.^26^	April 16	China	Correlation between Google Trends data and COVID-19 cases	Elsevier	International Journal of Infectious Diseases

In this paper, Google Trends data on the topic of “Coronavirus (virus)” in the United States are employed at both the national and state levels to explore the relationship between COVID-19 cases and deaths and online interest in the virus. First, a correlation analysis between Google Trends and COVID-19 data is performed; then, the role of Google Trends data in the predictability of COVID-19 is explored. To the best of our knowledge, this paper is the first attempt of this kind performed for the United States.

The rest of the paper is structured as follows. The Methods section details the data collection procedure and the statistical analysis tools and methods. The Results section consists of the correlation analysis and of the forecasting models at both national and state levels. The Discussion section presents the main findings of this work, along with the limitations of this paper and future research suggestions.

## Methods

Data from the Google Trends platform are retrieved in .csv^39^ and are normalized over the selected period. Google Trends reports the adjustment procedure as follows: “*Search results are normalized to the time and location of a query by the following process: Each data point is divided by the total searches of the geography and time range it represents to compare relative popularity. Otherwise, places with the most search volume would always be ranked highest. The resulting numbers are then scaled on a range of 0 to 100 based on a topic’s proportion to all searches on all topics. Different regions that show the same search interest for a term don't always have the same total search volumes*”^40^. The data collection methodology is designed based on the Google Trends Methodology Framework in Infodemiology and Infoveillance^41^. Note that the data may slightly vary based on the time of retrieval.

For keyword selection, the online interest in all commonly used variations is examined, and the variations are compared, i.e., “coronavirus (virus)”; “COVID-19 (search term)”; “SARS-COV-2 (search term)”; “2019-nCoV (search term)”; and “coronavirus (search term)”. Only “coronavirus (virus)” and “coronavirus (search term)” yield, as expected, considerably high online interest. Between the two, i.e., the topic (virus) and the search term, “coronavirus (virus)” is selected for further analysis.

Data on the worldwide distribution of COVID-19 cases and deaths are retrieved from Worldometer^9^. Data for the United States analysis of COVID-19 are retrieved from “The COVID Tracking Project”, which provides detailed structured data on COVID-19 cases and deaths nationally and at state level^10^. Maps of COVID-19 cases and deaths and online interest are created by the authors using the free online tools Pixelmap^42^ and Chartsbin^43^, with data from the respective sources^9,10^, while graphs, spider web charts, and maps of the correlation coefficients are created by the authors using Microsoft Excel (version 16.39).

As Google Trends data are normalized, the timeframe for which search traffic data are retrieved should exactly match the period for which COVID-19 data are available. Therefore, the timeframes for which analysis is performed are different among states, starting either on March 4th (for most cases) or on the date on which the first confirmed case was identified in each state, as shown in Table 2.

**Table 2:** Timeframes for which Google Trends data are retrieved by state.

March 4th–April 15th	USA; Arizona; California; Florida; Georgia; Illinois; Massachusetts; New Hampshire; New York; North Carolina; Oregon; Texas; Washington; Wisconsin
March 5th–April 15th	Nevada; New Jersey; Tennessee
March 6th–April 15th	Colorado; Indiana; Maryland; Pennsylvania
March 7th–April 15th	Hawaii; Kentucky; Minnesota; Nebraska; Oklahoma; Rhode Island; South Carolina; Utah
March 8th–April 15th	Connecticut; District of Columbia; Kansas; Missouri; Vermont; Virginia
March 9th–April 15th	Iowa; Louisiana; Ohio
March 11th–April 15th	Delaware; Michigan; New Mexico; South Dakota
March 12th–April 15th	Arkansas; Maine; Mississippi; Montana; North Dakota; Wyoming
March 13th–April 15th	Alabama; Alaska
March 14th–April 15th	Idaho
March 18th–April 15th	West Virginia

Each variable used in this study is divided by its full-sample standard deviation, estimated or calculated based on the basic formula of the standard deviation of a variable. By doing so, the inherent variability of each variable was moved, and thus, all variables have a standard deviation equal to 1. This equivalence makes it possible to compare the strength of the impact of the explanatory variables used on the dependent variable. The nonparametric^44^ unit root test is also applied to reveal whether or not the variables are stationary. The results suggest that both variables can be used directly in the present analysis without further transformation.

The first step in exploring the role of Google Trends in the predictability of COVID-19 is to examine the relationship between Google Trends and the incidence of COVID-19. As Pearson correlation analysis is the benchmark analysis in this kind of approach, the Pearson correlation coefficients (*r*) between the ratio (COVID-19 deaths)/(COVID-19 cases) and Google Trends data are calculated. In particular, a minimum variance bias-corrected Pearson correlation coefficient^45,46^ via a bootstrap simulation is applied to deal with the limited number of observations and, therefore, small sample estimation bias (also see^45,47^). The bias-corrected bootstrap coefficient ${\stackrel{\sim }{\rho }}^{b}$ for the Pearson correlation is given as follows:

$${\stackrel{\sim }{\rho }}^{b}={B}^{-1}\sum_{j=1}^{B}{\stackrel{\sim }{\rho }}_{j}^{b}\left(\rho \right)$$

where $B$ corresponds to the length of the bootstrap samples; in this case, it is set equal to 999^48^. Note that the terms “COVID-19 deaths” and “COVID-19 cases” refer to the cumulative (total) COVID-19 deaths and cases in the United States and that this terminology is used hereafter unless otherwise stated.

Next, secondary correlation analysis is performed using the Kendall rank correlation, which is a nonparametric test that measures the strength of dependence between two variables. The Kendall rank correlation is distribution free and is considered robust in ratio data. Considering two samples with sample sizes $n$, the total number of pairings is $\frac{1}{2}n(n-1)$. The following formula is used to calculate the value of the bias-corrected Kendall rank correlation:

$${\stackrel{\sim }{\tau }}^{b}={B}^{-1}\sum_{j=1}^{B}{\stackrel{\sim }{\tau }}_{j}^{b}\left(\tau \right)$$

where $\tau$ is given by $\tau =\frac{{n}_{c}- {n}_{d}}{\frac{1}{2}n(n-1)}$, ${n}_{c}$ is the concordant value, and ${n}_{d}$ is the discordant value.

Following, a COVID-19 predictability analysis approach based on Google Trends time series for the United States and all US states (plus DC) is performed. The predictability model is a quantile regression, which is considered to be a robust regression analysis against the presence of outliers in the sample; it was introduced by^49^. Building on the study conducted by^46^, a quantile regression that is bias corrected via balanced bootstrapping is employed. Such a model is the appropriate statistical approach for mitigating small sample estimation bias and the presence of outliers in the dataset, as it combines the advantages of bootstrap standard errors and the merits of quantile regression. Additional knowledge on quantile regression can be found in the studies conducted by^50^ and^51^, while recent applications of quantile regression can be found in^52,53^. More recently^54^ introduced unconditional quantile regression, while the study by^55^ provides further insights into robust estimates of regressions.

Let ${Y}_{t},$ with $t\in T$, be a time series that represents the dependent variable, supposing a bivariate specification. Quantile regression estimates the impact of the explanatory variable ${X}_{t}$, with $t\in T$, on the variable ${Y}_{t}$ at different points of the conditional $q$-quantile, with $q\in \left(\mathrm{0,1}\right)$, of the conditional distribution. A value of the $q$-quantile close to zero and a value of the $q$-quantile close to one represent the left (lower) and right (upper) tails of the conditional distribution, respectively. The conditional quantile function is defined as follows:

$$Q_{Y|X} \left( q \right) = {\text{X}}^{\prime } \beta_{q}$$

Given the distribution of ${Y}_{t}$, the estimation of the conditional quantile functions ${\beta }_{q}$ can be obtained by solving the following minimization problem:

$${\beta }_{q}=\mathrm{arg}\underset{\beta \in {\mathbb{R}}^{k}}{\mathrm{min}}E\left({\rho }_{q}\left(Y-X\beta \right)\right)$$

where ${\rho }_{q}\left(y\right)=y\left(q-{1}_{\left\{y<0\right\}}\right)$ represents the loss function.

By minimizing the sample analog $\left\{{y}_{1},\dots ,{y}_{n}\right\}$ that corresponds to a ${q}^{th}$ quantile sample, the estimator ${\beta }_{q}$ takes the following form:

$$\beta_{q} = {\text{arg}}\mathop {\min }\limits_{{\beta \in {\mathbb{R}}^{k} }} \mathop \sum \limits_{t = 1}^{n} \rho_{q} \left( {Y_{t} - X_{t}^{^{\prime}} \beta } \right) = {\text{arg}}\mathop {\min }\limits_{{\beta \in {\mathbb{R}}^{k} }} \left[ {q\mathop \sum \limits_{{Y_{t} \ge \beta X_{t} }} \left| {Y_{t} - \beta X_{t} } \right| + \left( {1 - q} \right)\mathop \sum \limits_{{Y_{t} < \beta X_{t} }} \left| {Y_{t} - \beta X_{t} } \right|} \right]$$

where $\beta {X}_{t}$ is an approximation of the conditional $q$-quantile of the variable ${Y}_{t}$.

In our analysis, ${Y}_{t}$ stands for the ratio (COVID-19 deaths)/(COVID-19 cases), ${\rm X}_{t-1}$ is the respective Google Trends value in lag order, and $t=1,\dots ,T$, with $T$ being the respective number of observations. A linear trend is used as well.

Finally, the bias-corrected parameter is estimated as follows:

$${\stackrel{\sim }{\beta }}^{b}\left(q\right)=\widehat{\beta }\left(q\right)-\widehat{bias}\left(\widehat{\beta }\left(q\right)\right)$$

where $\widehat{bias}\left(\widehat{\beta }\left(q\right)\right)$ is given by ${B}^{-1}{\sum }_{j=1}^{B}{\widehat{\beta }}_{j}^{*}\left(q\right)-\widehat{\beta }\left(q\right)$ and $q\in (0, 1)$ denotes the quantile considered and, in this case, is set equal to 0.5 (median). Median regression is considered more robust to outliers than, for example, least squares regression. Finally, it also avoids assumptions about the error parametric distribution^56^.

Αll estimation results reported in this paper were computed in the R programming environment^57^. In particular, we employed the R packages "quantreg" and "boot" to compute the quantile regression estimates and to perform the bootstrapping, respectively. The code is available in a “Supplementary Online Material file”.

## Results

**Figure 3:**
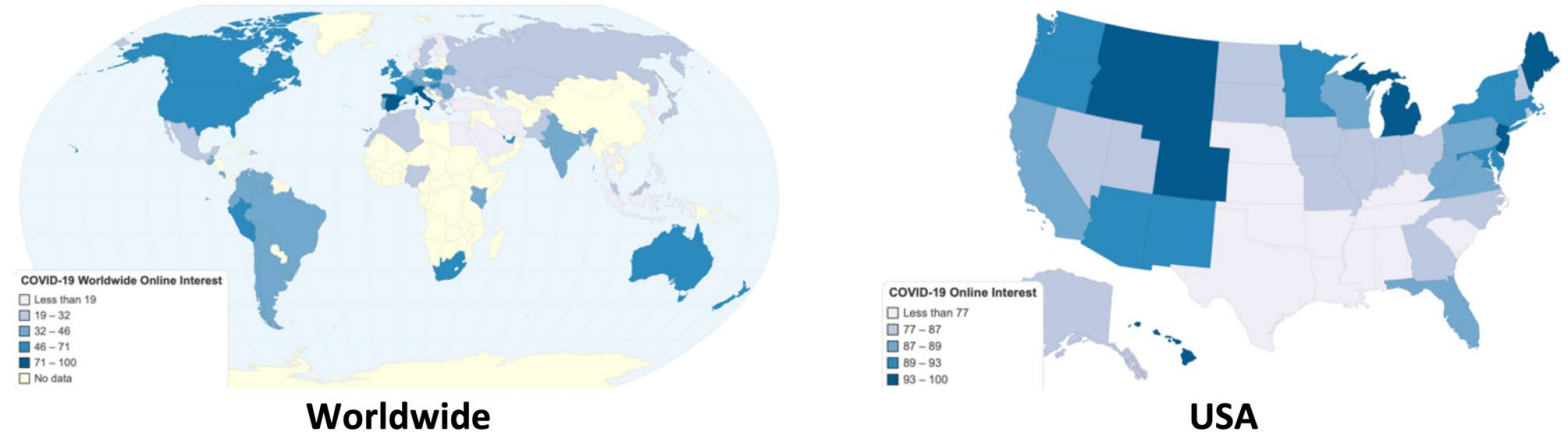
Heat maps of the worldwide and US online interest in “Coronavirus (Virus)” (Chartsbin^43^).

Figure 3 depicts the worldwide and US online interest in terms of Google queries in the “coronavirus (virus)” topic from January 22nd to April 15th, 2020. It shows that this topic is very popular, especially in Europe and North America. Specifically, interest in the United States is considerably high (above 70) for all US states.

To perform a first assessment of the relationship between Google Trends and COVID-19 data, the Pearson and Kendall rank correlations between the two variables are calculated, and the results are further compared. Tables 3 and 4 present the results of the Pearson and Kendall correlation analysis by state, respectively.

**Table 3:** Pearson correlation analysis by state.

State	Pearson correlation	Standard error	Wald test (*r* = 0)	*p*-value	State	Pearson correlation	Standard error	Wald test (*r* = 0)	*p*-value
USA	− 0.7054***	(0.0536)	[13.1672]	< 0.0001	Missouri	− 0.2627	(0.1608)	[1.6333]	0.1024
Alabama	− 0.6896***	(0.0748)	[9.2185]	< 0.0001	Montana	− 0.063	(0.1727)	[0.3651]	0.7151
Alaska	− 0.1162	(0.1276)	[0.9107]	0.3625	Nebraska	− 0.2763*	(0.1503)	[1.8381]	0.0661
Arizona	− 0.313**	(0.1292)	[2.4225]	0.0154	Nevada	− 0.3452**	(0.1519)	[2.273]	0.0230
Arkansas	0.4282***	(0.1105)	[3.8742]	0.0001	New Hampshire	− 0.406***	(0.1432)	[2.8349]	0.0046
California	− 0.4123***	(0.1300)	[3.1711]	0.0015	New Jersey	− 0.065	(0.2013)	[0.3227]	0.7469
Colorado	0.435**	(0.1761)	[2.4694]	0.0135	New Mexico	− 0.1474	(0.1367)	[1.0783]	0.2809
Connecticut	− 0.1266	(0.1895)	[0.668]	0.5041	New York	− 0.5925***	(0.0790)	[7.5016]	< 0.0001
Delaware	0.182	(0.2004)	[0.908]	0.3639	North Carolina	− 0.3172**	(0.1561)	[2.032]	0.0421
DC	− 0.3464**	(0.1632)	[2.1219]	0.0338	North Dakota	0.2567	(0.1705)	[1.5056]	0.1322
Florida	− 0.3171**	(0.1559)	[2.034]	0.0420	Ohio	− 0.1645	(0.1979)	[0.8311]	0.4059
Georgia	− 0.3467**	(0.1462)	[2.3708]	0.0178	Oklahoma	− 0.1703	(0.1713)	[0.9944]	0.3200
Hawaii	− 0.1591	(0.1692)	[0.9405]	0.3470	Oregon	0.4605***	(0.1432)	[3.2154]	0.0013
Idaho	0.0614	(0.1436)	[0.4276]	0.6689	Pennsylvania	− 0.3645**	(0.1446)	[2.5218]	0.0117
Illinois	0.2501*	(0.1512)	[1.6541]	0.0981	Rhode Island	− 0.0366	(0.1805)	[0.2031]	0.8391
Indiana	0.0162	(0.1884)	[0.086]	0.9314	South Carolina	− 0.2094	(0.1400)	[1.4958]	0.1347
Iowa	− 0.2172	(0.1539)	[1.4112]	0.1582	South Dakota	0.3518*	(0.1920)	[1.8323]	0.0669
Kansas	0.1141	(0.1748)	[0.6531]	0.5137	Tennessee	− 0.3878***	(0.1495)	[2.5937]	0.0095
Kentucky	− 0.2789*	(0.1663)	[1.677]	0.0935	Texas	0.0223	(0.1931)	[0.1157]	0.9079
Louisiana	− 0.2422	(0.1713)	[1.4141]	0.1573	Utah	− 0.2135	(0.1448)	[1.4749]	0.1402
Maine	− 0.1811	(0.1387)	[1.3062]	0.1915	Vermont	− 0.3255**	(0.1549)	[2.1007]	0.0357
Maryland	− 0.0385	(0.2045)	[0.1884]	0.8505	Virginia	− 0.286**	(0.1414)	[2.0228]	0.0431
Massachusetts	− 0.4285***	(0.1421)	[3.0152]	0.0026	Washington	− 0.5805***	(0.0835)	[6.9492]	< .0001
Michigan	− 0.1045	(0.1757)	[0.5949]	0.5519	West Virginia	0.0033	(0.0426)	[0.0781]	0.9378
Minnesota	− 0.3513**	(0.1550)	[2.2657]	0.0235	Wisconsin	− 0.3972***	(0.1285)	[3.09]	0.002
Mississippi	0.308	(0.1975)	[1.5599]	0.1188	Wyoming	0.396**	(0.1840)	[2.1524]	0.0314

*p < 0.1; **p < 0.05; ***p < 0.01.

**Table 4:** Kendall rank correlation analysis by state.

State	Kendall correlation	Standard error	Wald test (r = 0)	*p*-value	State	Kendall correlation	Standard error	Wald test (*r* = 0)	*p*-value
USA	− 0.6230***	(0.0780)	[7.9891]	1.36E−15	Missouri	− 0.2919**	(0.1187)	[2.4585]	0.0140
Alabama	− 0.0679	(0.1389)	[0.4887]	0.6251	Montana	− 0.2903**	(0.1405)	[2.0660]	0.0388
Alaska	− 0.2713**	(0.1279)	[2.1218]	0.0339	Nebraska	− 0.3589***	(0.1216)	[2.9517]	0.0032
Arizona	− 0.3372**	(0.1313)	[2.5684]	0.0102	Nevada	− 0.2989**	(0.1424)	[2.0996]	0.0358
Arkansas	0.4083***	(0.1497)	[2.7278]	0.0064	New Hampshire	− 0.3397***	(0.1313)	[2.5884]	0.0096
California	− 0.2801**	(0.1285)	[2.1794]	0.0293	New Jersey	− 0.0690	(0.1451)	[0.4759]	0.6342
Colorado	0.0510	(0.1459)	[0.3498]	0.7265	New Mexico	− 0.2851**	(0.1184)	[2.4070]	0.0161
Connecticut	− 0.3060**	(0.1371)	[2.2320]	0.0256	New York	− 0.4379***	(0.0871)	[5.0283]	0.0000
Delaware	− 0.0095	(0.1545)	[0.0618]	0.9507	North Carolina	− 0.2817**	(0.1305)	[2.1582]	0.0309
DC	− 0.4986***	(0.1119)	[4.4565]	0.0000	North Dakota	0.2737*	(0.1507)	[1.8160]	0.0694
Florida	− 0.3247**	(0.1323)	[2.4538]	0.0141	Ohio	− 0.4007***	(0.1350)	[2.9683]	0.0030
Georgia	− 0.3262**	(0.1290)	[2.5291]	0.0114	Oklahoma	− 0.2902**	(0.1400)	[2.0725]	0.0382
Hawaii	− 0.2372*	(0.1262)	[1.8805]	0.0600	Oregon	0.2751**	(0.1320)	[2.0830]	0.0373
Idaho	− 0.1065	(0.1435)	[0.7425]	0.4578	Pennsylvania	− 0.4173***	(0.1192)	[3.5013]	0.0005
Illinois	− 0.1379	(0.1369)	[1.0077]	0.3136	Rhode Island	− 0.1088	(0.1497)	[0.7266]	0.4675
Indiana	− 0.0738	(0.1344)	[0.5491]	0.5830	South Carolina	− 0.1900	(0.1172)	[1.6215]	0.1049
Iowa	− 0.4162***	(0.1172)	[3.5507]	0.0004	South Dakota	− 0.1255	(0.1641)	[0.7645]	0.4446
Kansas	− 0.0851	(0.1480)	[0.5752]	0.5651	Tennessee	− 0.3333***	(0.1236)	[2.6974]	0.0070
Kentucky	− 0.3496***	(0.1275)	[2.7423]	0.0061	Texas	0.0202	(0.1346)	[0.1502]	0.8806
Louisiana	− 0.3701***	(0.1345)	[2.7529]	0.0059	Utah	− 0.3029***	(0.1138)	[2.6617]	0.0078
Maine	− 0.3012**	(0.1388)	[2.1690]	0.0301	Vermont	− 0.3658***	(0.1298)	[2.8179]	0.0048
Maryland	− 0.2630**	(0.1301)	[2.0218]	0.0432	Virginia	− 0.4270***	(0.1141)	[3.7409]	0.0002
Massachusetts	− 0.3833***	(0.1377)	[2.7829]	0.0054	Washington	− 0.4560***	(0.0909)	[5.0152]	0.0000
Michigan	− 0.3908***	(0.1466)	[2.6658]	0.0077	West Virginia	− 0.0733	(0.1126)	[0.6515]	0.5147
Minnesota	− 0.3785***	(0.1383)	[2.7372]	0.0062	Wisconsin	− 0.3506***	(0.1191)	[2.9441]	0.0032
Mississippi	0.0992	(0.1486)	[0.6679]	0.5042	Wyoming	− 0.0416	(0.1481)	[0.2811]	0.7786

*p < 0.1; **p < 0.05; ***p < 0.01.

As reported in Table 3, statistically significant correlations are observed for the United States and for the states of Alabama, Arkansas, California, Colorado, Florida, Georgia, Illinois, Kentucky, Massachusetts, Minnesota, Nebraska, Nevada, New Hampshire, New York, North Carolina, Oregon, Pennsylvania, South Dakota, Tennessee, Vermont, Virginia, Washington, Wisconsin, and Wyoming as well as DC. The states of Iowa, Louisiana, Maine, Mississippi, Missouri, North Dakota, South Carolina, and Utah do not marginally reach the *p* < 0.1 threshold of statistical significance, i.e., $p\in (0.1, 0.2)$.

**Figure 4:**
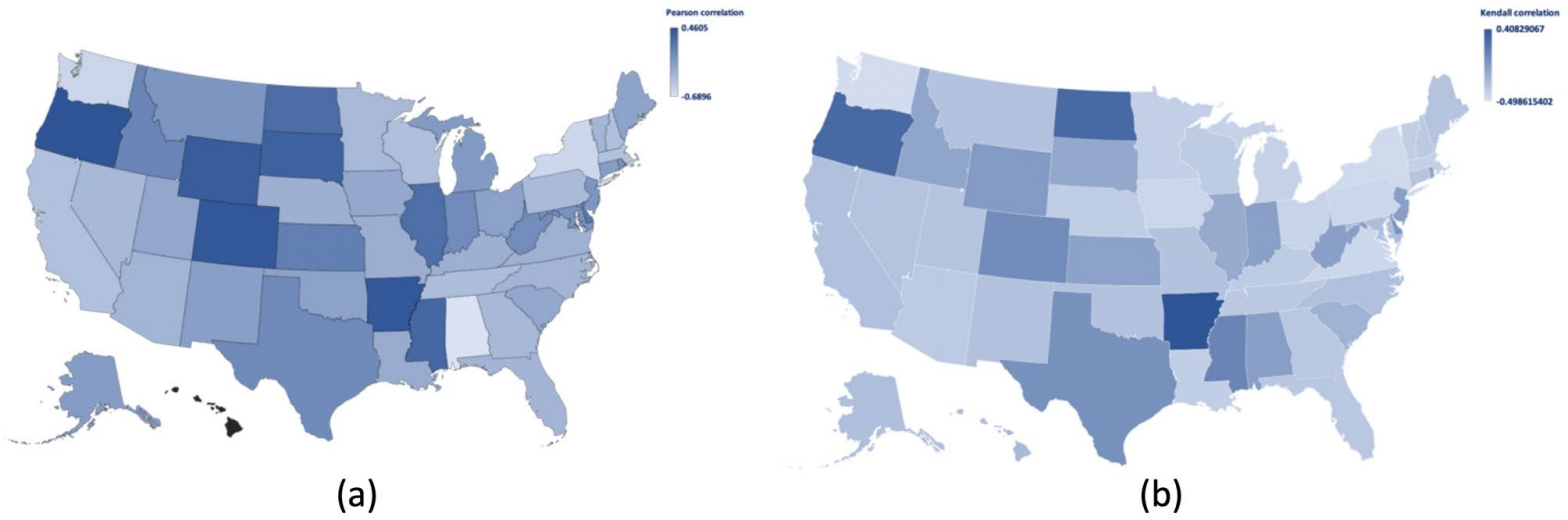
Heat map of the (**a**) Pearson and (**b**) Kendall correlation coefficients by state (Microsoft Excel).

Based on the Kendall correlation analysis, statistically significant correlations are observed for the United States and for the states of Alaska, Arizona, Arkansas, California, Connecticut, Florida, Georgia, Hawaii, Iowa, Kentucky, Louisiana, Maine, Maryland, Massachusetts, Michigan, Minnesota, Missouri, Montana, Nebraska, Nevada, New Hampshire, New Mexico, New York, North Carolina, North Dakota, Ohio, Oklahoma, Oregon, Pennsylvania, Tennessee, Utah, Vermont, Virginia, Washington, and Wisconsin as well as DC. Figure 4 depicts the heat map of the (a) Pearson and (b) Kendall correlation coefficients in the United States by state over the period examined.

**Figure 5:**
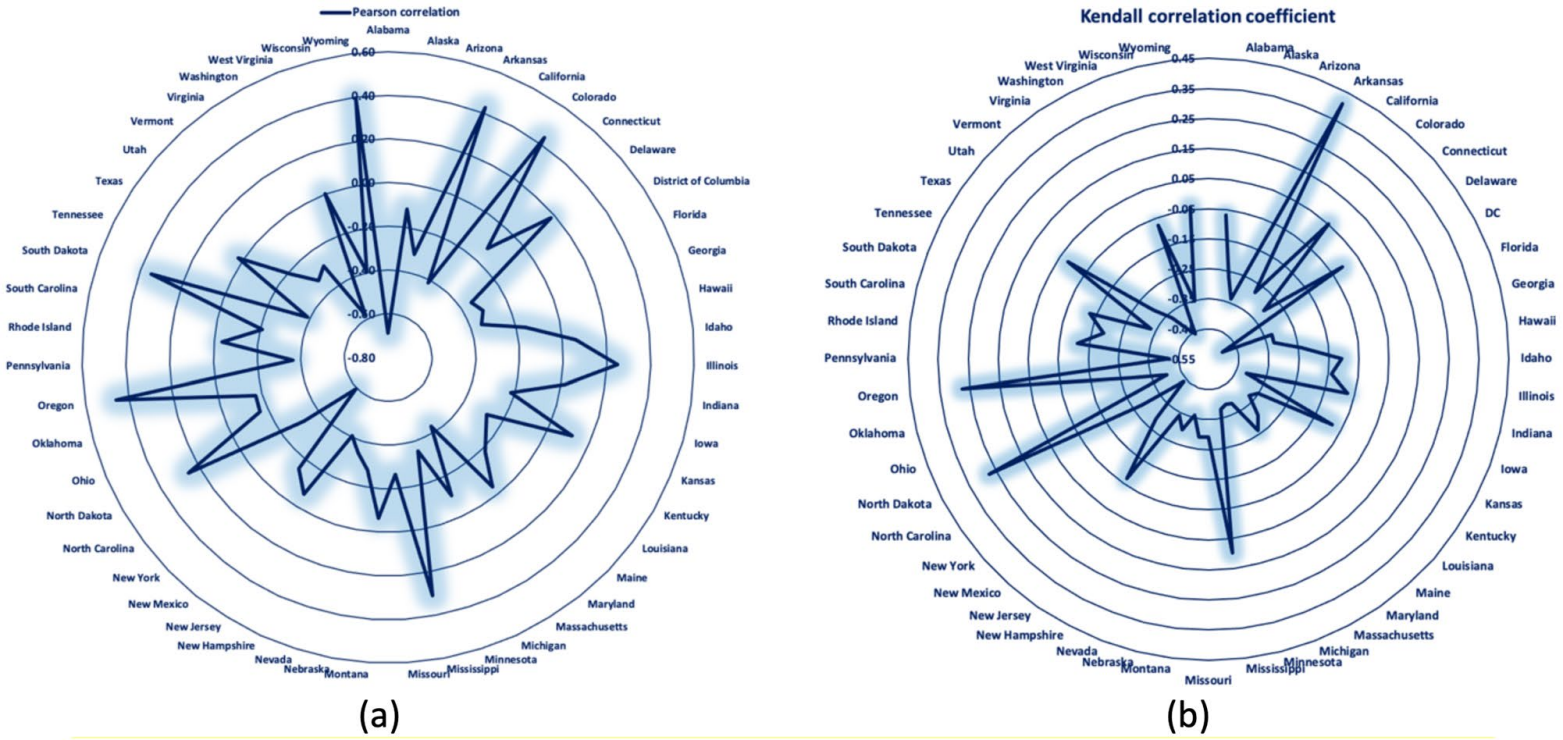
Radar chart of the (**a**) Pearson and (**b**) Kendall correlation coefficients by state (Microsoft Excel).

As depicted in the heat maps and in the spider web charts for the respective correlation analyses in Fig. 5, visual comparison of the two approaches indicates that the results are consistent in both analyses.

**Figure 6:**
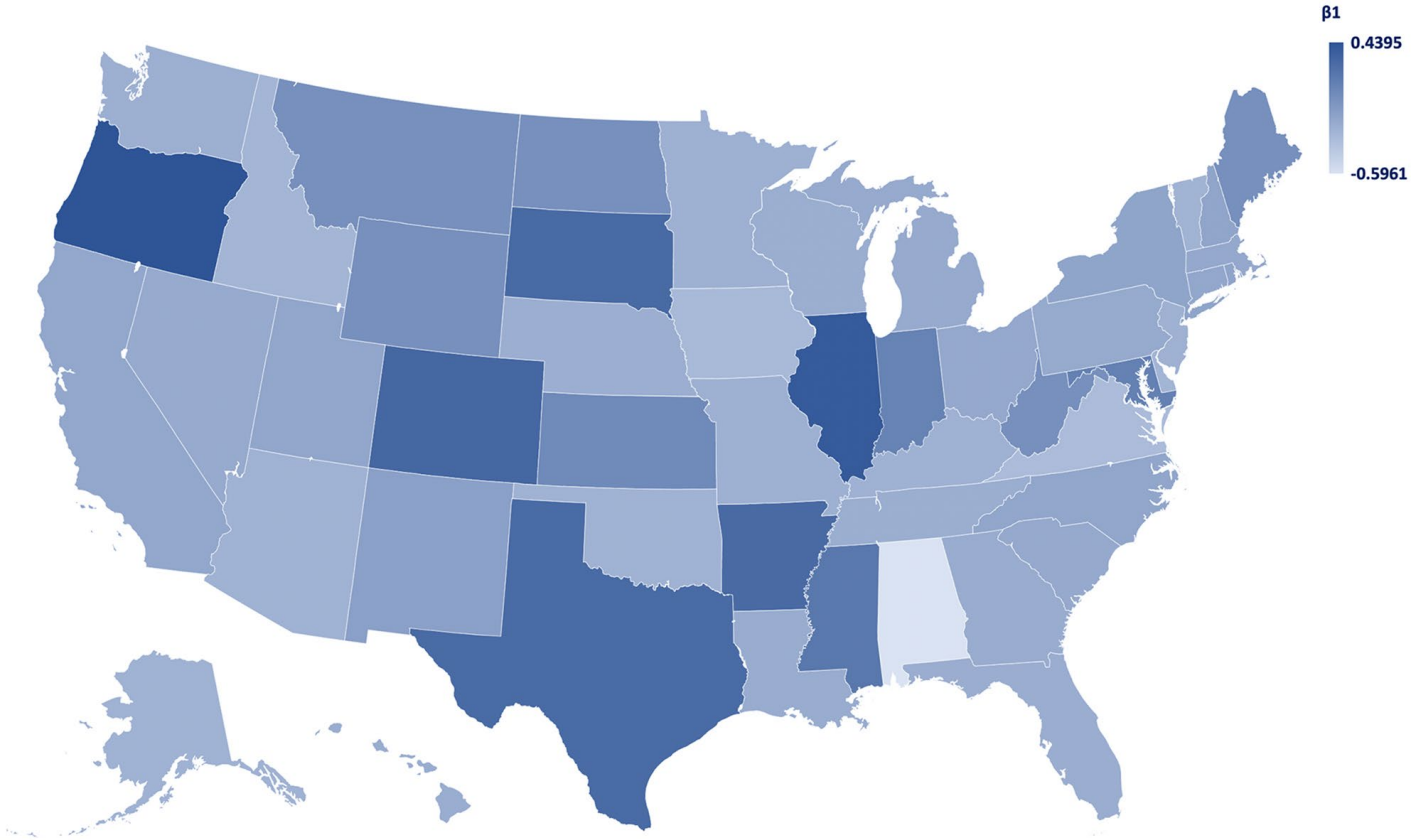
Heat map of ${\beta }_{1}$ of the predictability analysis models by state (Microsoft Excel).

However, the main purpose of this study is to explore the predictability of COVID-19 using Google Trends data in the United States. Proceeding with the results of the predictability analysis, Fig. 6 depicts the heat map for ${{\varvec{\beta}}}_{1}$ by state, while Table 5 presents the quantile regression estimated predictability models for the US and for each US state (plus DC). As shown, the estimated Google Trends models exhibit strong COVID-19 predictability.

**Table 5:** Predictability analysis by state.

	${\beta }_{0}$			${\beta }_{1}$			${\beta }_{2}$		
USA	− 0.0509	(0.4339)	[− 0.1172]	− 0.7506***	(0.2197)	[− 3.4173]	− 0.0014	(0.0169)	[− 0.0831]
AL	0.8944***	(0.2176)	[4.1099]	− 0.5961***	(0.1160)	[− 5.1383]	− 0.0413***	(0.0070)	[− 5.8850]
AK	− 1.4528***	(0.2003)	[− 7.2539]	− 0.2449**	(0.1006)	[− 2.4341]	0.0663***	(0.0087)	[7.6030]
AZ	− 1.4183***	(0.1309)	[− 10.8362]	− 0.2429***	(0.0817)	[− 2.9745]	0.0637***	(0.0049)	[12.8777]
AR	− 0.2565	(0.4658)	[− 0.5507]	0.2785	(0.2531)	[1.1004]	0.0023	(0.0124)	[0.1825]
CA	− 1.4274***	(0.0936)	[− 15.2521]	− 0.1634***	(0.0539)	[− 3.0325]	0.0642***	(0.0046)	[13.8481]
CO	− 0.9688***	(0.1916)	[− 5.0561]	0.3007	(0.2587)	[1.1623]	0.0290***	(0.0074)	[3.9132]
CT	− 1.7866***	(0.0654)	[− 27.3353]	− 0.1645***	(0.0470)	[− 3.4989]	0.0782***	(0.0026)	[30.6221]
DE	− 2.0415***	(0.4639)	[− 4.4003]	− 0.2687	(0.2446)	[− 1.0987]	0.0715***	(0.0110)	[6.4873]
DC	− 1.3077***	(0.1980)	[− 6.6064]	− 0.1548*	(0.0849)	[− 1.8228]	0.0578***	(0.0094)	[6.1513]
FL	− 1.5483***	(0.0766)	[− 20.2209]	− 0.2128***	(0.0431)	[− 4.9412]	0.0715***	(0.0024)	[29.3170]
GA	− 1.5727***	(0.0808)	[− 19.4690]	− 0.2047***	(0.0570)	[− 3.5898]	0.0721***	(0.0042)	[17.2658]
HI	− 1.6732***	(0.0873)	[− 19.1647]	− 0.2083***	(0.0470)	[− 4.4343]	0.0758***	(0.0041)	[18.3027]
ID	− 1.8929***	(0.1465)	[− 12.9167]]	− 0.2686***	(0.0663)	[− 4.0507]	0.0866***	(0.0067)	[12.8631]
IL	− 1.4466***	(0.1404)	[− 10.3063]	0.3943***	(0.0707)	[5.5764]	0.0680***	(0.0056)	[12.2022]
IN	− 1.4674***	(0.2157)	[− 6.8020]	0.0977	(0.1624)	[0.6018]	0.0693***	(0.0065)	[10.7392]
IA	− 1.5912***	(0.1402)	[− 11.3507]	− 0.2957***	(0.0733)	[− 4.0346]	0.0732***	(0.0042)	[17.3342]
KS	− 1.5579***	(0.2298)	[− 6.7799]	0.0463	(0.1101)	[0.4204]	0.0635***	(0.0106)	[5.9774]
KY	− 1.5530***	(0.1396)	[− 11.1222]	− 0.2415***	(0.0599)	[− 4.0291]	0.0719***	(0.0062)	[11.5292]
LA	− 1.6432***	(0.0602)	[− 27.2763]	− 0.2050***	(0.0357)	[− 5.7381]	0.0751***	(0.0026)	[28.6534]
MD	− 1.1066***	(0.2339)	[− 4.7306]	0.1135	(0.1008)	[1.1255]	0.0550***	(0.0088)	[6.2834]
MA	− 1.6424***	(0.0771)	[− 21.3061]	− 0.1757***	(0.0538)	[− 3.2668]	0.0742***	(0.0034)	[21.8651]
MI	− 1.7657***	(0.0813)	[− 21.7133]	− 0.1884***	(0.0406)	[− 4.6375]	0.0800***	(0.0032)	[25.2349]
MN	− 1.6085***	(0.0773)	[− 20.7963]	− 0.2344***	(0.0521)	[− 4.4970]	0.0728***	(0.0027)	[26.9966]
MS	− 1.3047***	(0.2959)	[− 4.4088]	0.1773	(0.1600)	[1.1086]	0.0570***	(0.0082)	[6.9200]
MO	− 1.5382***	(0.0883)	[− 17.4271]	− 0.2326***	(0.0478)	[− 4.8610]	0.0718***	(0.0051)	[14.0987]
NE	− 1.4875***	(0.1909)	[− 7.7908]	− 0.2192***	(0.0746)	[− 2.9375]	0.0717***	(0.0063)	[11.3935]
NV	− 1.6778***	(0.0862)	[− 19.4683]	− 0.1872***	(0.0348)	[− 5.3846]	0.0763***	(0.0037)	[20.4946]
NH	− 1.6586***	(0.0723)	[− 22.9526]	− 0.1515***	(0.0365)	[− 4.1562]	0.0741***	(0.0025)	[30.0037]
NJ	− 1.8518***	(0.2428)	[− 7.6277]	− 0.2395	(0.2427)	[− 0.9867]	0.0688***	(0.0060)	[11.3949]
NM	− 1.2414***	(0.1640)	[− 7.5679]	− 0.1188	(0.0803)	[− 1.4805]	0.0593***	(0.0066)	[8.9371]
NY	− 1.2201***	(0.0468)	[− 26.0596]	− 0.1482***	(0.0562)	[− 2.6358]	0.0482***	(0.0043)	[11.2916]
NC	− 1.6575***	(0.0953)	[− 17.3914]	− 0.1613***	(0.0476)	[− 3.3848]	0.0722***	(0.0038)	[18.8471]
OH	− 1.8408***	(0.1464)	[− 12.5751]	− 0.1758**	(0.0750)	[− 2.3436]	0.0790***	(0.0048)	[16.3817]
OK	− 1.7038***	(0.0544)	[− 31.2986]	− 0.2463***	(0.0318)	[− 7.7497]	0.0767***	(0.0026)	[29.5090]
OR	− 0.7953***	(0.2019)	[− 3.9392]	0.4395***	(0.1362)	[3.2257]	0.0293***	(0.0069)	[4.2697]
PA	− 1.3917***	(0.1279)	[− 10.8769]	− 0.1845**	(0.0758)	[− 2.4348]	0.0716***	(0.0041)	[17.5561]
RI	− 1.4924***	(0.0752)	[− 19.8418]	− 0.1461***	(0.0408)	[− 3.5844]	0.0588***	(0.0049)	[12.1036]
SC	− 1.2889***	(0.0941)	[− 13.7030]	− 0.1816***	(0.0513)	[− 3.5395]	0.0520***	(0.0069)	[7.5216]
SD	− 1.1230***	(0.2939)	[− 3.8212]	0.2815**	(0.1388)	[2.0277]	0.0537***	(0.0084)	[6.4280]
TN	− 1.5098***	(0.0658)	[− 22.9294]	− 0.2157***	(0.0524)	[− 4.1179]	0.0676***	(0.0020)	[33.1730]
TX	− 1.4766***	(0.3041)	[− 4.8557]	0.2749	(0.1903)	[1.4442]	0.0660***	(0.0077)	[8.5342]
UT	− 1.4381***	(0.1399)	[− 10.2768]	− 0.1586**	(0.0723)	[− 2.1944]	0.0720***	(0.0069)	[10.3640]
VT	− 1.5359***	(0.1854)	[− 8.2848]	− 0.2499***	(0.0848)	[− 2.9476]	0.0770***	(0.0081)	[9.5352]
VA	− 1.5878***	(0.2504)	[− 6.3400]	− 0.3147***	(0.1021)	[− 3.0837]	0.0767***	(0.0106)	[7.2484]
WA	− 1.3476***	(0.1540)	[− 8.7488]	− 0.2236**	(0.1007)	[− 2.2212]	0.0660***	(0.0101)	[6.5118]
WI	− 1.3407***	(0.0992)	[− 13.5142]	− 0.2143***	(0.0698)	[− 3.0711]	0.0618***	(0.0053)	[11.6287]

The numbers in parentheses report the standard errors; the t-statistics are given in brackets.

***, ** and * indicate statistical significance at the 0.01, 0.05 and 0.1 levels, respectively. The corresponding critical values are 2.575, 1.96 and 1.645.

Note that due to the low number of observations, the states of Maine, Montana, North Dakota, West Virginia, and Wyoming are not included in the predictability analysis results, but they are given the value “zero (0)” to be included in the heat map for purposes of uniformity.

## Discussion

As of July 29th, 2020, there were 16,920,857 COVID-19 recorded cases worldwide, with the reported death toll at 664,141 and the number of recovered patients at 10,485,316^9^. In light of the COVID-19 pandemic and to find new ways of forecasting the spread of the disease, infodemiology approaches have provided valuable input in monitoring and forecasting the development of the COVID-19 pandemic over time and in measuring and analyzing the public’s awareness and response. Google Trends and Twitter have been identified as the most popular infodemiology sources, while other social media, such as Facebook and Instagram, exhibit promising results in analyzing users’ online behavioral patterns^13^.

Social media platforms can provide us with more qualitative data that can shift the focus to other directions. Such approaches include sentiment analysis, educational purposes, and efforts to measure and raise public awareness. Recent approaches to analyzing aspects of the COVID-19 pandemic using social media data include monitoring the Twitter usage of G7 leaders^58^, monitoring self-reported symptoms on Twitter^59^, and analyzing the public perception of the disease through Facebook^60^. Moreover, infodemiology sources have provided valuable input in recruiting online survey participants through Facebook to measure individuals’ COVID-19 confidence levels^61^ and in assessing the behavioral variations in COVID-19-related online search traffic in more than one search engine^62^. Finally, commentaries that make recommendations on the integration of other social media platforms, such as Facebook, Reddit, and TikTok, for disseminating medical information to inform public health and policy have been published^63^.

Google Trends offers a solid foundation for quantitative analysis with respect to the monitoring and predictability of COVID-19, as in the analysis presented in this study, where Google Trends data on the “coronavirus (virus)” topic were used to explore the predictability of COVID-19 in the United States at both national and state level. First, for a preliminary assessment of the relationship between Google Trends and COVID-19 data, Pearson correlation and Kendall rank correlation analyses were performed. Statistically significant correlations were observed for the United States and for several US states, which is in line with previous studies that argue that there is a relationship between Google Trends and COVID-19 data.

The COVID-19 predictability analysis, which used a quantile regression approach, exhibits very promising results and indicates the most important contribution of this study to the international literature: detecting and predicting the early spread of COVID-19 at the regional level. This contribution can be a substantial supplement in further assisting local authorities in taking the appropriate measures to handle the spread of the disease.

**Figure 7:**
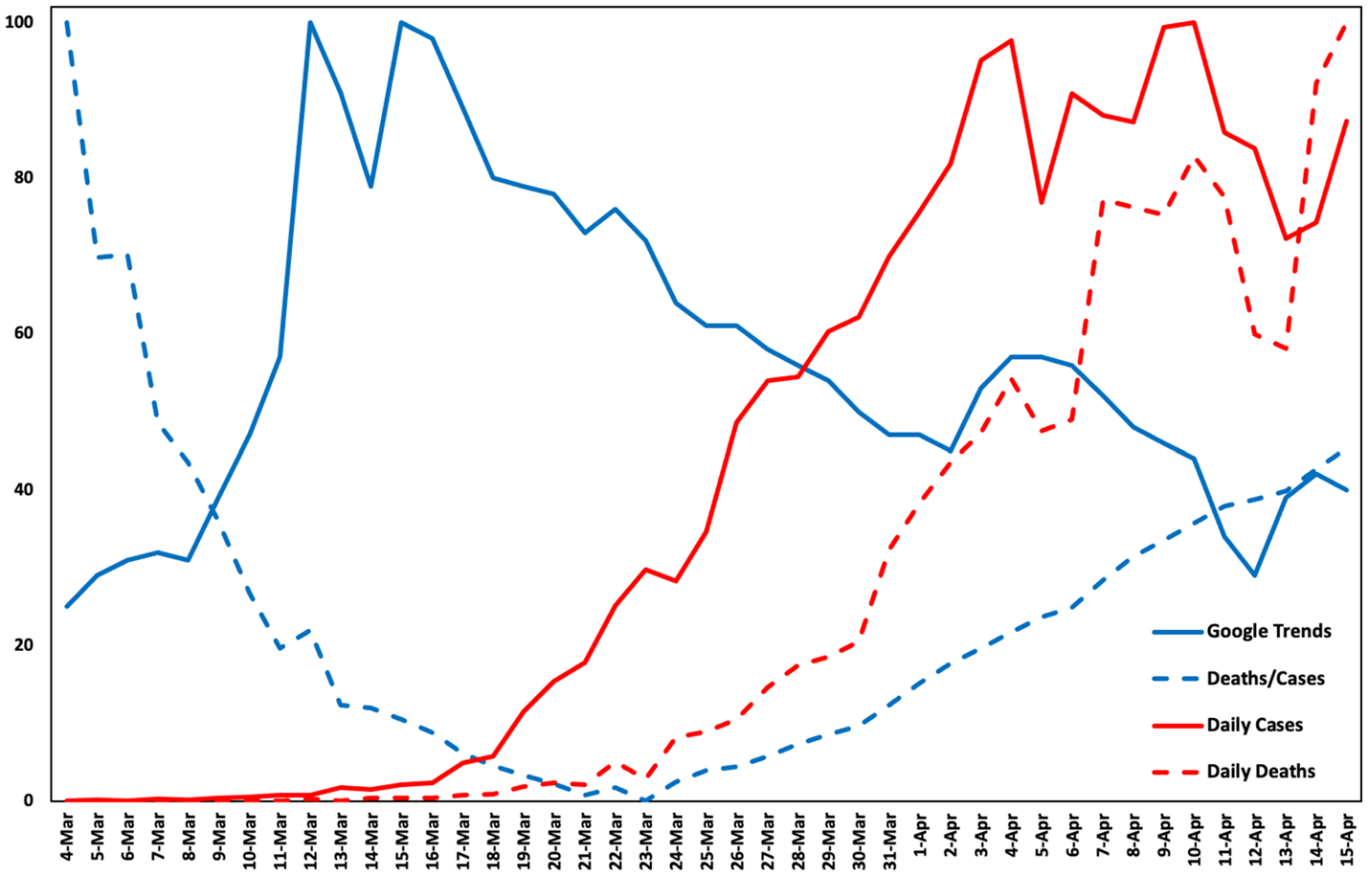
COVID-19 and Google Trends data from March 4th to April 15th in the US (Microsoft Excel).

Figure 7 illustrates a graph of the COVID-19 deaths/cases ratio, daily COVID-19 deaths, daily COVID-19 cases, and the respective Google Trends normalized data in the United States from March 4th to April 15th, 2020. For purposes of consistency in the graph, the COVID-19-related time series are normalized on a 0–100 scale. As depicted in the graph and confirmed by the predictability analysis, the two variables are not linearly dependent. Instead, they exhibit an inversely proportional relationship, meaning that as COVID-19 progresses, the online interest in the virus decreases.

From a behavioral point of view, this result can be explained as follows. First, online interest starts to increase and reaches a peak as the number of confirmed cases becomes high and as the deaths rates start to show that the pandemic does indeed have severe consequences. However, after a certain period, the interest has an inverse course, which could also indicate that the public is overwhelmed by information overload and decreases its information “intake”. The spike in Google queries and the decline in the ratio of COVID-19 deaths/cases could be attributed to the spread of the virus over these days and the “delay” in deaths. Regarding this latter point, this means that cases increase while the total number of deaths has not yet started to considerably increase.

The latter point is in line with previous work on the topic^27^ suggesting that although significant correlations between COVID-19 and Google data are observed, the relationship tends to decrease in both strength and significance in regions that have been affected by COVID-19 as we move forward in time because the interest in the virus decreases. This decrease is counterintuitive and occurs before the case and death curves start to exhibit a downward trend, i.e., when a region is being heavily affected, independent of whether or not it has reached its peak. However, it would be interesting for future investigators to explore the relationship from this point onwards since, as shown in Fig. 7, the lines converge, with this convergence being indicative of a future change in the relationship dynamics when deaths peak at a later point and when they start their downward course as well.

The above can partly explain the differences in signs among states in both the Pearson and Kendall rank correlation coefficients, but a more in-depth explanation from a statistical perspective is that the Pearson correlation coefficient is estimated as the average of the deviations of observations from the sample mean. The weights of observations in the tails of the distribution are equal to the weight of other observations, and therefore, the outliers could affect the estimation of the results, especially in the case of the small sample. In consideration of ties, this study employs a bootstrap bias-corrected approach, but the main conclusions are based on quantile regressions. Unlike linear measures of dependency, quantile regression is considered superior in a sampling situation and more resistant to outliers than linear regressions, the Pearson correlation, or the Kendall rank correlation^64^. Taking into account that the current pandemic is a dynamic process that constantly evolves and has a serious social impact, it is very probable that there now exist—or, at a later stage, could develop—several data anomalies (e.g., due to non-pharmaceutical interventions); therefore, formal statistical tools such as the Pearson and Kendall rank correlations should be carefully interpreted.

This study has limitations. First, data from only one search engine are considered. Although Google Trends is the most popular search engine, some data on the coronavirus topic from other search engines were not included in this analysis. Second, the data at this point are very limited, and the results are based on few observations. Third, the 50 (+ 1) states exhibit diversity in terms of confirmed cases and deaths. Therefore, any conclusions drawn from this analysis refer to each case individually. Despite the known limitations of online search traffic data, the use of infodemiology metrics for informing public health and policy in general and for monitoring outbreaks and epidemics in particular has received wide attention.

To dynamically find the determinants of COVID-19, the predictability analysis in this study provides insights into how online search traffic data can play a considerable role in forming public health policies, especially in times of epidemics and outbreaks, when real-time data are essential. With the COVID-19 pandemic, the world is in uncharted territory socially, economically, and socially. This situation calls for immediate action and open research and data, and the term “multidisciplinary” has never before been more important. To that end, the role of big data in providing “*opportunities for performing modeling studies of viral activity and for guiding individual country healthcare policymakers to enhance preparation for the outbreak*” has been acknowledged^65^, and current research on the subject should focus on both exploring the role of other infodemiology variables in the predictability of COVID-19 and combining infodemiology sources with traditional sources to explore the full potential of what online real-time data have to offer for disease surveillance.

## Supplementary information

Supplementary Information.

## Data Availability

The COVID-19 and query datasets analyzed during the current study are available on the COVID-19 Tracking Project website^10^ and on the “Google Trends” explore page^39^, respectively.
