# Supplementary material for: COVID-19 predictability in the United States using Google Trends time series
Source: Sci Rep. 2020 Nov 26;10:20693. doi: 10.1038/s41598-020-77275-9 (PMC7692493; doi:10.1038/s41598-020-77275-9)
Supplement: Supplementary file 1 — Supplementary Information. [file 41598_2020_77275_MOESM1_ESM.pdf]

Supplementary Material for

"COVID-19 predictability in the United States using Google Trends time series"

Amaryllis Mavragani[1,*] and Konstantinos Gkillas[2]
[1]*Department of Computing Science and Mathematics, Faculty of Natural Sciences, University of Stirling, Stirling, FK9 4LA, Scotland, UK*
[2]*Department of Business Administration, University of Patras, Patras, Greece*; gillask@upatras.gr
*amaryllis.mavragani1@stir.ac.uk

This file consists of the functions and the programs used in the statistical analysis.

```
############################################################################################
############################################################################################
############################################################################################
############################################################################################
############################################################################################
############################################################################################
# Author: Dr. Konstantinos Gkillas, University of Patras
# Email: gillask@upatras.gr
# Project: Exploring COVID-19 predictability in USA by analyzing Google Trends time series

############################################################################################
############################################################################################
############################################################################################
############################################################################################
############################################################################################
############################################################################################

# Functions
bcorrelation<-function(data,ind){
# Change type="pearson" for Pearson correlation
  x<-data[ind,1]
  y<-data[ind,2]
    m <- cbind(x, y)
    rc<-cor(m, method="kendall", use="pairwise")
     ro<-rc[1,2]
         return(ro)
     }

 convert.z.score<-function(zB, one.sided=NULL) {
         if(is.null(one.sided)) {
           pval = pnorm(-abs(zB));
            pval = 2 * pval
            } else if(one.sided=="-") {
               pval = pnorm(zB);
                } else {
                  pval = pnorm(-zB);
                }
                   return(pval);
       }

   bs <- function(data,ind,formula) {
   d <- data[ind,]
    fit <- rq(formula, tau=T, data=d)
     return(coef(fit))
   }
############################################################################################
############################################################################################
############################################################################################
############################################################################################
############################################################################################
############################################################################################
############################################################################################
############################################################################################
############################################################################################
############################################################################################
############################################################################################
############################################################################################

############################################################################################
############################################################################################
############################################################################################
############################################################################################
############################################################################################
############################################################################################
#End

```

```r
###########################################################################################
###########################################################################################
###########################################################################################
###########################################################################################
###########################################################################################
###########################################################################################
# Author: Dr. Konstantinos Gkillas, University of Patras
# Email: gillask@upatras.gr
# Project: Exploring COVID-19 predictability in USA by analyzing Google Trends time series

###########################################################################################
###########################################################################################
###########################################################################################
###########################################################################################
###########################################################################################
###########################################################################################

# Language used by R to display the error messages
 Sys.setenv(LANG = "en")
# Suppresion of all data in the R memory
 rm(list=ls())
# Clear the console in RStudio
 cat("\014")
# R packages used in the program and the functions
library(xlsx)
library(Hmisc)
library(goft)
library(Hmisc)
library(boot)
library(quantreg)
# To be changed by the user
  date_execution = "00_00_00"
# Starting date
# The duration of the program is proportional to the number of bootstrap simulations
  starting_time = Sys.time()
 Time <- paste("Starting time of the process:", starting_time)
    write(Time, file=Time, append = TRUE) # Writing in the same file (use of "Append = TRUE")
     write("", file=Time, append = TRUE)
#####################################################################################################
#####################################################################################################
#####################################################################################################
#####################################################################################################
#####################################################################################################
#####################################################################################################
#Read bivariate data as a matrix
 attach(Dataset)
#y Depedent variable e.g. Total Deaths/Total Cases
#x Explanatory variable e.g. Google trends
     #x<-(x-mean(x))/sd(x)
   #y<-(y-mean(y))/sd(y)
  #Check also the number of lags needed
  xm<-as.matrix(xm)
# Define the number of replications
    R1=999
#####################################################################################################
#####################################################################################################
#####################################################################################################
#####################################################################################################
#####################################################################################################
#####################################################################################################
# Bias corrected correlations
    tbnvarpi<- boot(xm, statistic=bcorrelation, R=R1,sim = "balanced")
     z1<-tbnvarpi$t
  bnvarpi<-mean(z1)
# Change type="pearson"
    rcc<-cor(xm, method="kendall", use="pairwise")
     roo<-rcc[1,2]
# Bias corrected estimators
      bpC<-(2*roo)-bnvarpi
       spC<-sd(z1)

# Wald Test Null, r=0
        w1<-abs(bpC/spC)
           p_Value_w1<-convert.z.score(w1, one.sided=NULL)

#####################################################################################################
#####################################################################################################
#####################################################################################################
#####################################################################################################
#####################################################################################################
#####################################################################################################
#Bias corrected regressions
T<-0.5
Bootreg <-boot(data=Dataset,statistic=bs,
# Numer of lags considered
               formula=y~y1+x,R=R1,sim = "balanced")
  b0<-Bootreg$t0[1]
   b1<-Bootreg$t0[2]
    b2<-Bootreg$t0[3]
```

```
#Bias corrected estimators
  b0C<-(2*b0)-mean(Bootreg$t[,1])
   b1C<-(2*b1)-mean(Bootreg$t[,2])
    b2C<-(2*b2)-mean(Bootreg$t[,3])
  sb0C<-sd(Bootreg$t[,1])
   sb1C<-sd(Bootreg$t[,2])
    sb2C<-sd(Bootreg$t[,3])
#T-tests
  T0<-b0C/sb0C
   T1<-b1C/sb1C
    T2<-b2C/sb2C
####################################################################################################
####################################################################################################
####################################################################################################
####################################################################################################
####################################################################################################
####################################################################################################
####################################################################################################
####################################################################################################
####################################################################################################
####################################################################################################
####################################################################################################
####################################################################################################
#End
```
